# PPA1 Deficiency Causes a Deranged Galactose Metabolism Recognizable in Neonatal Screening

**DOI:** 10.3390/metabo13111141

**Published:** 2023-11-10

**Authors:** Melanie T. Achleitner, Judith J. M. Jans, Laura Ebner, Johannes Spenger, Vassiliki Konstantopoulou, René G. Feichtinger, Karin Brugger, Doris Mayr, Ron A. Wevers, Christian Thiel, Saskia B. Wortmann, Johannes A. Mayr

**Affiliations:** 1 University Children’s Hospital, Salzburger Landeskliniken (SALK), Paracelsus Medical University, 5020 Salzburg, Austria; me.achleitner@salk.at (M.T.A.); la.ebner@salk.at (L.E.); j.spenger@salk.at (J.S.); r.feichtinger@salk.at (R.G.F.); kar.brugger@salk.at (K.B.); d.mayr@salk.at (D.M.); s.wortmann@salk.at (S.B.W.); 2 Department of Genetics, University Medical Center Utrecht, Utrecht University, 3584 CX Utrecht, The Netherlands; j.j.m.jans@umcutrecht.nl; 3 Center for Molecular Medicine, University Medical Center Utrecht, Utrecht University, 3584 CX Utrecht, The Netherlands; 4 Department of Pediatrics, Austrian Newborn Screening, Medical University of Vienna, 1090 Vienna, Austria; vassiliki.konstantopoulou@meduniwien.ac.at; 5 Department of Human Genetics, Translational Metabolic Laboratory, Donders Institute for Brain, Cognition and Behaviour, Radboud University Medical Center, 6525 GA Nijmegen, The Netherlands; ron.wevers@radboudumc.nl; 6 Center for Child and Adolescent Medicine, Pediatrics I, University Heidelberg, Analysezentrum 3, 69120 Heidelberg, Germany; christian.thiel@med.uni-heidelberg.de; 7 Amalia Children’s Hospital, Radboudumc, 6525 GA Nijmegen, The Netherlands

**Keywords:** PPA1, pyrophosphate, galactosemia, galactose, hyperbilirubinemia, inborn metabolic disorder

## Abstract

Two siblings showed increased galactose and galactose-related metabolites in neonatal screening. Diagnostic workup did not reveal abnormalities in any of the known disease-causing enzymes involved in galactose metabolism. Using whole-exome sequencing, we identified a homozygous missense variant in *PPA1* encoding the cytosolic pyrophosphatase 1 (PPA1), c.557C>T (p.Thr186Ile). The enzyme activity of PPA1 was determined using a colorimetric assay, and the protein content was visualized via western blotting in skin fibroblasts from one of the affected individuals. The galactolytic activity of the affected fibroblasts was determined by measuring extracellular acidification with a Seahorse XFe96 analyzer. PPA1 activity decreased to 22% of that of controls in the cytosolic fraction of homogenates from patient fibroblasts. PPA1 protein content decreased by 50% according to western blot analysis, indicating a reduced stability of the variant protein. The extracellular acidification rate was reduced in patient fibroblasts when galactose was used as a substrate. Untargeted metabolomics of blood samples revealed an elevation of other metabolites related to pyrophosphate metabolism. Besides hyperbilirubinemia in the neonatal period in one child, both children were clinically unremarkable at the ages of 3 and 14 years, respectively. We hypothesize that the observed metabolic derangement is a possible mild manifestation of PPA1 deficiency. Unresolved abnormalities in galactosemia screening might result in the identification of more individuals with PPA1 deficiency, a newly discovered inborn metabolic disorder (IMD).

## 1. Introduction

An inborn metabolic disorder (IMD) is defined as an impairment of a specific enzyme or the disruption of a biochemical pathway which is intrinsic to the pathomechanism [1]. Currently, the Online Mendelian Inheritance in Man database (OMIM, https://www.omim.org/) lists at least 1629 genes in which pathogenic variants have been associated with an IMD (access on 29 March 2023).

**Figure 1:**
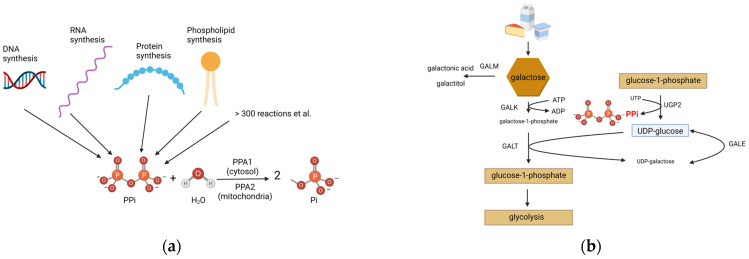
Sites of cellular PPi formation and details of galactose metabolism. (**a**) The majority of PPi is produced through four pathways: DNA, RNA, protein and phospholipid synthesis. Many more pathways contribute to the PPi concentration in the cell. PPi is hydrolyzed by differently localized PPA (PPA1/PPA2) to two molecules of Pi. (**b**) UDP-glucose is produced out of glucose-1-phosphate, which produces PPi. UDP-glucose is necessary for the breakdown of galactose, which is further metabolized in glycolysis. Abbreviations: UTP, uridine diphosphate; PPi, pyrophosphate; PPA, pyrophosphatases; Pi, orthophosphate. This figure was created with BioRender.com.

Some IMDs are amenable or even preventable with (early) dietary treatment and are therefore included in neonatal screening programs worldwide according to the Wilson and Jungner criteria, which have been revised by the World Health Organization (WHO) [2]. Many neonatal screening programs identify classical galactosemia (MIM#230400) by measuring total galactose and galactose-1-phosphate uridyltransferase (GALT) activity in dried blood spots (DBSs). In human metabolism, galactose is converted to galactose-1-phosphate, which is then epimerized to glucose-1-phosphate before entering the glycolytic pathway for energy production (Figure 1). For epimerization, UDP-glucose donates UDP. The production of the essential UDP-glucose for the metabolism of galactose results in the formation of inorganic pyrophosphate (PPi) [3].

Generally, human cells produce PPi in all fundamental pathways, like DNA, RNA, protein and phospholipid synthesis. The Kyoto Encyclopedia of Genes and Genomes (KEGG, https://www.genome.jp/kegg/ (accessed on 29 March 2023)) database lists approximately 300 reactions in human metabolism which generate PPi. (Figure 1) Cellular PPi is hydrolyzed into two molecules of orthophosphate (Pi) by the enzyme pyrophosphatase (PPA). The hydrolysis of PPi into Pi is required for the synthesis of adenosine triphosphate (ATP) either by mitochondrial oxidative phosphorylation or, to a lesser extent, in glycolysis. In humans, like in other eukaryotes, rapid hydrolysis of PPi in the cytosol is catalyzed by PPA1. Mitochondria have their own pyrophosphatase, PPA2 [4]. Biallelic pathogenic variants in *PPA2* have recently been associated with sudden cardiac death with a bimodal distribution in manifestation which peaks in the first 2 years of life, followed by an additional peak in adolescence, with the latter presumably associated with modest consumption of alcohol (MIM# 617223) [5]. *PPA1* has not yet been found to be related to monogenic human disease, but there are association studies in cancer [6]. The role of PPA1 was also studied in Ppa1 knockout mice, with the conclusion that biallelic loss of function (LoF) variants in *PPA1* are embryonic lethal. The same study also showed that PPA1 might have a function regulating glucose tolerance and insulin resistance in adipose tissue [7]. 

Here, we characterize two siblings who were flagged in neonatal screening for galactosemia. Exome sequencing revealed a homozygous variant in *PPA1* leading to decreased enzyme activity of PPA1, abnormalities in galactose metabolism, and an increase in other metabolites related to PPi formation.

## 2. Materials and Methods

### 2.1. Neonatal Screening

DBSs (filter paper (Schleicher & Schuell 903, Dassel, Germany)) were collected with a heel stick between 36 and 72 h of life during regular national neonatal screening in Austria (https://www.meduniwien.ac.at/hp/en/newborn-screening/ (accessed on 23 March 2023)) [8,9].

### 2.2. Genetic Testing

Targeted sequencing for known galactosemia-causing genes was performed with either a TruSight One Sequencing Panel (Illumina) (for *GALE*, *GALT* and *GALK1*) or Sanger sequencing (for *GALM*). Exome sequencing (ES) was performed in leucocyte-derived DNA of the female individual in strict compliance with all necessary ethical and institutional protocols, and written informed consent was obtained from the parents. ES was carried out in accordance with standard protocols, validated capture, sequencing and variant calling pipelines [10,11,12]. Sanger sequencing was used to confirm the homozygous *PPA1* variant in both individuals using standard protocols using the following primers: PPA1-Ex7-fwd 5′-TCCGTGATAAGTCATTCCACAG-3′′ and PPA1-Ex7-rev 5′-CATTTCCCCAAAGCCTGATA-3′. 

### 2.3. Sample Preparation for Untargeted Metabolomics

Samples from the affected individuals, II.1 and II.3, and the heterozygous father, I.1, were extracted from DBS cards (3 mm, ~3.1 μL of whole blood) with the addition of 140 μL of methanol with stable-isotope-labeled internal standards, followed by a twenty-minute ultra-sonication step. DBS samples were diluted with 60 μL of 0.3% formic acid and filtered using a methanol-preconditioned 96-well filter plate (Acro prep, 0.2 μm GHP, NTRL, 1 mL well; Pall Corporation, Ann Arbor, MI, USA) and a vacuum manifold. The filtrate was collected in a 96-well plate (Advion, Ithaca, NY, USA). 

### 2.4. Untargeted Metabolomics

Untargeted metabolomics using direct-infusion high-resolution mass spectrometry was performed as described previously [13]. Briefly, extracts from DBSs (13 µL) were injected in triplicate into the Q-Exactive high-resolution mass spectrometer using a TriVersa NanoMate system (Advion, Ithaca, NY, USA) controlled by Chipsoft software (version 8.3.3, Advion). The Q-Exactive high-resolution mass spectrometer was operated in positive- and negative-ion mode with automatic polarity switching. There were two time segments of 1.5 min, with a total run time of 3.0 min. The scan range was a 70 to 600 mass-to-charge ratio (*m*/*z*), with a resolution of 140,000 at *m*/*z* = 200. Data acquisition was performed using Xcalibur software (Thermo Scientific™, Waltham, MA, USA). Using MSConvert15 (ProteoWizard Software Foundation, Palo Alto, CA, USA), raw data files containing scanning time, *m*/*z* and peak intensity were converted to mzXML format. Data processing and peak calling were carried out using an in-house-developed pipeline. 

### 2.5. Data Analysis of Untargeted Metabolomics

Detected mass peaks were annotated by matching the *m*/*z* of the mass peak with a range of five parts per million to metabolite masses present in the Human Metabolome Database (HMDB, www.hmdb.ca (accessed on 23 March 2023)) Metabolite annotations without adduct ions in negative or positive mode ([M − H]^−^, [M + H]^+^) or with the single adduct ions [M + Na]^+^, [M + K]^+^ and [M + Cl]^−^ were selected. For each sample, the intensities of these five mass peaks were summed, resulting in one (summed) mass peak intensity per metabolite annotation, for ~6600 summed mass peaks in total. Next, exogenous and drug metabolite annotations were excluded, resulting in ~1875 summed mass peaks in total, corresponding to ~380 metabolite annotations, since mass peaks can account for several isomers. For each mass peak per patient sample, the deviation from the intensities in 30 within-run control samples was indicated with a Z-score, calculated as follows: Z-score = (intensity patient sample − mean intensity control samples)/standard deviation intensity control samples. For elevated metabolites, the corresponding metabolic pathways were identified using HMDB and KEGG.

### 2.6. Cell Culture

Primary human skin fibroblasts were collected through skin biopsy from one of the affected individuals (II.1) and the father (I.1, heterozygous carrier). Control fibroblasts were obtained from other patients with either monogenic or infectious disorders not influencing PPA function or galactose metabolism. 

The cells were cultured in high-glucose DMEM medium (D5648, Sigma-Aldrich, St. Louis, MO, USA). The medium was supplemented with 10% fetal calf serum (FCS), sodium pyruvate and antibiotics. The fibroblasts were passaged with a splitting ratio of 1:3. For experiments, semi-confluent cultures were used at passages between 4 and 10 [14,15].

### 2.7. 10,000× g Supernatant from Primary Human Skin Fibroblasts

Fibroblasts were cultured to 80% confluency in three 180 cm^2^ flasks. The medium was removed, and cells were washed with 10 mL of 1× phosphate-buffered saline (PBS). The cells were trypsinized with 10 mL of 0.05% trypsin/0.53 mM EDTA for 3 min. The cell suspension was collected in 50 mL tubes and 3 mL of FCS was added. The cells were centrifuged at 1300 rpm for 5 min. The supernatant was discarded, and the cell pellet was resuspended in 1 mL of 1× PBS. The pellet was transferred into an Eppendorf tube and centrifuged at 600× *g* 4 °C for 5 min. The cell pellet was washed twice with 1 mL of 1× PBS. The pellet was weighted and resuspended in 10 times the amount of 10 mM Tris used, pH 7.4. The cells were disrupted with a tight-fitting Potter-Elvehjem-Homogenisator. One fifth of the volume of the cell suspension of 1.5 M sucrose was rapidly added and mixed, and the homogenate was centrifuged at 600× *g* 4 °C for 10 min. The supernatant was transferred into a new Eppendorf tube and centrifuged at 10,000× *g* 4 °C for 10 min. Aliquots of the 10,000× *g* supernatant were stored at −80 °C for further analysis. 

### 2.8. Quantification of PPA Activity in 10,000× g Supernatants

Enzyme activity measurement of PPA1 was carried out in 10,000× *g* supernatant from the fibroblasts of individuals II.1 and I.1 and controls [16]. Two independent measurements at different PPi concentrations ranging from 0 to 0.2 mM were performed. The formation of Pi from PPi was detected via colorimetric absorption measurement as described previously [17]. The measurement was normalized according to the total protein amount in the samples, as measured with a bicinchoninic acid (BCA) protein assay. The contamination of PPA2 in 10,000× *g* supernatant was determined via western blotting of the 10,000× *g* supernatant and whole-cell lysates. 

### 2.9. Western Blot Analysis

Western blot determination of PPA1 derived from 10,000× *g* supernatant from the fibroblasts of II.1, I.1 and controls was performed in two independent blots with standard procedures using a rabbit polyclonal anti-PPA1 antibody (14985-1-AP, Proteintech, Rosemont, IL, USA) and a rabbit monoclonal anti-PPA2 antibody (cat# ab177935, Abcam, Cambridge, UK) [5]. As a loading control, the rabbit polyclonal glyceraldehyde-3-phosphate dehydrogenase (GAPDH) antibody (2275-PC-100, Szabo Scandic, Vienna, Austria) was used. Whole-cell lysates of different fibroblasts were stained with the rabbit monoclonal anti-PPA2 antibody. Loading was normalized according to the total protein amount in the samples, as measured via BCA. Membranes were exposed for 10 min. For statistical analysis of two independent blots, a *t*-test was carried out.

### 2.10. Enzyme Activity Measurement of Recombinant PPA1

Complementary DNA (cDNA) of the *PPA1* variant and *PPA1* wild type was cloned into the plasmid pRSETB (ThermoFisher, Waltham, MA, USA). PPA1 was expressed and purified as previously described [5]. Protein concentration was adjusted via Ponceau S staining and western blotting (Figure S2). Enzyme activity was measured at different PPi concentrations ranging from 0 mM to 0.2 mM with colorimetric end-point detection of phosphate and ammonium heptamolybdate at 620 nm in a 96-well plate. Phosphate production was calculated using a standard curve from 0.075 to 5 nmol phosphate [17]. For statistical analysis of two independent enzyme activity measurements, a one-way ANOVA was carried out.

### 2.11. Extracellular Flux Assay of Primary Human Skin Fibroblasts

Primary human skin fibroblasts of II.1, I.1 and controls were shifted from high-glucose DMEM medium to high-galactose medium [18]. The cells were grown in 75 cm^2^ flasks to 80% confluency. The medium was discarded, and the cells were washed with 5 mL 1× PBS. The cells were detached with 5 mL of 0.05% trypsin/0.53 mM EDTA for 3 min at 37 °C. The cell suspension was neutralized in 10 mL of high-galactose medium. The tubes were centrifuged for 5 min at 2000 rpm. The cell pellet was resuspended in 4 mL of high-galactose medium, and the cells were counted with a CASY cell counter. The cells were diluted to 150,000 cells/mL, and 80 µL was seeded into 96-well Seahorse plate (Agilent, Santa Clara, CA, USA). The plate was incubated for 1 h under laminar flow for a uniform cell layer. After that, the plate was placed in the incubator at 37 °C and 5% CO_2_ overnight. The sensor cartridge was calibrated with 200 µL of H_2_O at 37 °C without CO_2_ in an incubator overnight. Additionally, 50 mL of XF Calibrant was also incubated at 37 °C without CO_2_. The next day, the sensor cartridge was hydrated with 200 µL of XF Calibrant and the cartridge was placed in the incubator at 37 °C without CO_2_ for 1 h. For the glycolytic stress test, 200 mM L-glutamine was added to 50 mL of Agilent Seahorse medium, pH 7.4. The following inhibitors/uncoupler were prepared: 0.01 µM galactose (port A), 5 µM oligomycin (port B) and 0.05 µM 2-deoxy-D-glucose (port C). The cell plate was washed two times with 120 µL of the prepared Seahorse medium, and finally, 180 µL of Seahorse medium was added per well. The cell plate was placed in the incubator at 37 °C without CO_2_ for 1 h. After adding the inhibitors/uncoupler to the sensor cartridge, the calibration step commenced. The baseline measurement comprised 4 cycles, which included 2 min of mixing, 2 min of waiting and 5 min of measuring for each cycle. After each injection, 4 measuring points were recorded, which also comprised 2 min of mixing, 2 min of waiting and 5 min of measuring for each cycle. The galactolytic capacity was calculated according to the glycolytic capacity: $ECAR\;after\;addition\;of\;oligomycin-\left(non-galactolytic\;acidification\right)=galactolytic\;capacity$. For statistical analysis of two independent extracellular flux analyses, a one-way ANOVA was carried out. 

### 2.12. Determination of UDP-Gal and UDP-Glc Levels via UPLC-PDA

Determination of sugar nucleotides was performed in the fibroblasts of patient II.3 and in the fibroblasts of the father (I.1), as described previously [19].

### 2.13. Isoelectric Focusing of Serum Transferrin and Alpha-1-Antitrypsin

Isoelectric focusing (IEF) of transferrin was performed on patients’ and controls’ sera on a PhastSystem (GEHealthcare), as described in [20].

### 2.14. LC-MS Analysis of Whole-Serum N-Glycans

LC-MS analysis of N-glycans from whole-serum glycoproteins of a control pool (*n* = 120) and the PMM2-CDG patients was conducted using the GlycoWorks RapiFluor-MS N-Glykan-Kit (Waters, Milford, MA, USA) on an Integrated UPLC-FLR/QTOF mass spectrometry system with integrated software (BioAccord, Waters, Milford, MA, USA), as described in [21].

## 3. Results

### 3.1. Clinical Information

**Figure 2:**
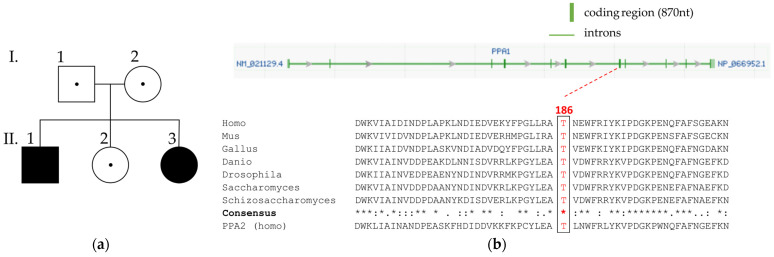
Pedigree of the family, PPA1 variant and conservation of affected amino acid position. (**a**) Pedigree structure of an Austrian family with non-consanguineous parents. The oldest boy (II.1) and the youngest girl (II.3) were homozygous for the PPA1 variant. (**b**) Genomic structure and location of the PPA1 variant (GenBank NM_021129.4). The green-filled boxes show exons, and the green lines indicate intronic regions. The red line shows the position of the missense variant. Phylogenetic analysis of PPA1 is shown by multiple sequence alignment carried out with Clustal Omega algorithm. PPA2 (GenBank NM_176869.3), nt: nucleotides, *: conservation of amino acids.

The boy (II.1) was the first and the girl (II.3) the third child born to non-consanguineous Austrian parents. Both parents as well as the second child had no medical concerns, and they did not show abnormalities in galactose metabolism. The family history was negative, especially for unexplained disabilities or death. (Figure 2) Both children were born after normal pregnancy and delivery and had an unremarkable postnatal adaptation, with the exception of transient hyperbilirubinemia with elevated liver transaminases treated with phototherapy (for 4 days) in the girl. 

Both children were developing and thriving age-appropriately without any medical concerns until the ages of 3 and 14 years. Ophthalmological investigations were unremarkable. No clinical symptoms of glucose homeostasis problems were reported.

### 3.2. Genetic Investigations

Targeted sequencing (and ES) of all known galactosemia-causing genes (*GALK1*, *GALT*, *GALE*, *GALM*) did not show any relevant (likely) pathogenic variants or variants of unknown significance (VUS) (American College of Medical Genetics & Genomics (ACMG) criteria [22]. Exome analysis investigating all captured human genes revealed a homozygous missense variant in *PPA1* (NM_021129.3) at position c.557C>T (p.Thr186Ile) in exon 7, which was rated as likely pathogenic (PM2: 2, PS3: 4). We are aware that candidate genes usually have no ACMG rating and therefore have to be classified as VUS when applying the ACMG rating for established disease genes [23]. (Figure 2) The Genome Aggregation Database (gnomAD, https://gnomad.broadinstitute.org/ (accessed on 23 March 2023)) does not list the identified variant, c.557C>T (p.Thr186Ile), or any other homozygous missense variants in PPA1. The PPA1 variant was shown to segregate with disease using Sanger sequencing. (Figure S1) Phylogenetic analysis of PPA1 showed a high conservation of threonine at amino acid position 186 in higher eukaryotes. The affected amino acid position was also conserved in the corresponding mitochondrial PPA (PPA2), which supports pathogenic relevance of the identified PPA1 variant (Figure 2).

### 3.3. Metabolic Investigations

**Figure 3:**
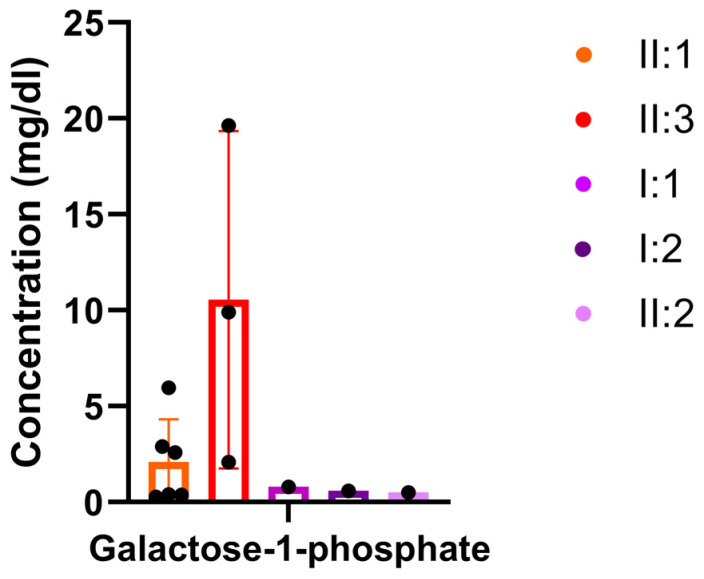
Galactose-1-phosphate in blood of the affected individuals and heterozygous family members. The affected individuals showed elevated galactose-1-phosphate, and the boy showed 3 normal values (reference values: 0.2–0.8 mg/dL) (II.1), of which 2 were documented during a galactose-restricted diet. Erythrocyte galactose-1-phosphate in classic galactosemia is usually >10 mg/dL. Galactose-1-phosphate was measured at least 3 times over a period of at least half a year.

Both children showed elevated total galactose (ref. 0–20 mg/dL; II.1: 33.6 mg/dL; II.3: 23.1 mg/dL) and galactose-1-phosphate (ref. 0.2–0.8 mg/dL; II.1: 2.6 mg/dL; II.3: 19.6 mg/dL) with normal GALT activity (ref. >4 Units/g Hemoglobin (Hb); II.1: 12.76 Units/g Hb; II.3: 8.9 Units/g Hb) in neonatal screening. While galactose levels were normalized in follow-up measurements after 2 months in the girl, galactose-1-posphate was repeatedly elevated in both affected individuals during follow-up (Figure 3). During a galactose-restricted diet, the affected boy (II.1) showed a decrease in galactose-1-phosphate levels (Figure 3, Table S1). He also had normal galactose-1-phosphate levels in the follow-up; unfortunately, during this time, the diet was not exactly documented. In the investigation of polyols and sugars from urine, the boy (II.1) had increased galactose at 31 mmol/mol creatinine (normal range 0–18 mmol/mol creatinine) at the age of 11 years (Table S2). In the affected girl, galactitol was elevated at 85 mmol/mol creatinine (normal range: 10–63 mmol/mol creatinine) in the urine at the age of 4 months (Table S3). All values of standard metabolic and other clinical chemistry investigations are shown in Tables S1–S3. IEF of serum transferrin and alpha-1-antitrypsin to assess the presence of congenital disorders of glycosylation as well as LC-MS analysis of whole-serum N-glycans revealed normal values for both patients.

Untargeted metabolomics of the blood samples of II.1, II.3 (affected siblings) and I.1 (heterozygous father) revealed 1881 features, of which 19 were elevated in both affected individuals (defined as Z-score > 2.0). Through pathway analysis of the metabolites found to be increased, 13 of these were found to be linked to known intracellular metabolic pathways. The remaining six metabolites were not related to any known metabolic pathways and likely resulted from nutrition, muscle degradation, blood regulation or protein degradation (Table 1). Further pathway analysis of these 13 elevated metabolites revealed that 6 of them take part in pathways which can lead to PPi formation. Among the metabolites found to be increased, we identified galactose-1-phosphate and galactitol of galactose metabolism, metabolites from amino acid metabolism (isobutyrylglycine, aminoadipic acid, imidazole acetol-phosphate, N-acetylglutamine, N6-acetyl-L-lysine, N-acetyl-L-glutamate 5-semialdehyde), fatty acid metabolism (8-[(aminomethyl)sulfanyl]-6-sulfanyloctanoic acid) and nucleotide metabolism (pyrimidine).

### 3.4. Determination of PPA1 and PPA2 Protein Amount via Western Blotting

**Figure 4:**
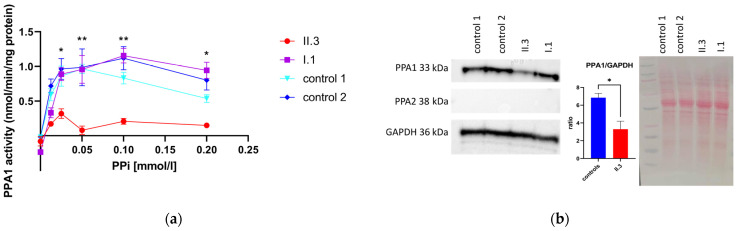
Enzyme activity of primary human skin fibroblasts derived from the affected individual (II.3), father (I.1) and controls. (**a**) Enzyme activity of PPA1 in 10,000× *g* supernatant of primary human skin fibroblasts of one affected individual (II.3), heterozygous father (I.1) and controls. The enzyme activity was determined at different pyrophosphate concentrations ranging from 0.0125 mM to 0.2 mM. At least five replicates in two independent measurements were performed for each PPi concentration. Error bars show the standard error of the mean (SEM). Enzyme activity at 0.1 mM PPi concentration. For the patient, mean: 0.21, SEM: 0.1; for the controls, mean: 1.03, SEM: 0.3; (**b**) western blotting of 10,000× *g* supernatant of primary human skin fibroblasts. For loading control, the GAPDH antibody was used. Densitometry of PPA1 was measured in two independent western blots in comparison to the loading control, GAPDH (number of measurements: affected individual (II.3): *n* = 2, controls: *n* = 6). The western blot was analyzed with an unpaired *t*-test. * *p* < 0.05, ** *p* < 0.01, SD: standard deviation.

PPA1 content was similar in I.1 (heterozygous father) and the controls. In the sample of the affected individual II.3, the PPA1 level was reduced to 48% of that of the controls (Figure 4). PPA2 was not detectable via western blotting in the 10,000× *g* supernatant of any sample, indicating that there was no appreciable contamination of mitochondrial matrix protein in the preparations of 10,000× *g* supernatant (Figure 4). To evaluate the efficiency of the removal of mitochondria in 10,000× *g* samples, we determined the levels of PPA2 in whole-cell lysates of different fibroblasts (Figure S2).

### 3.5. Pyrophosphatase Activity in Primary Human Skin Fibroblasts

In the heterozygous father and controls, PPA1 activity increased with higher PPi concentrations (Figure 4). At 0.1 mM PPi, the highest activity in the heterozygous father and control 2 was observed. The enzyme activity in the sample of the affected individual did not increase with PPi concentration, as observed in the controls and heterozygous father. The enzyme activity in the affected individual was reduced to 22% of that of controls at a 0.1 mM substrate concentration (Figure 4).

### 3.6. Enzyme Activity Measurement of Recombinant PPA1

**Figure 5:**
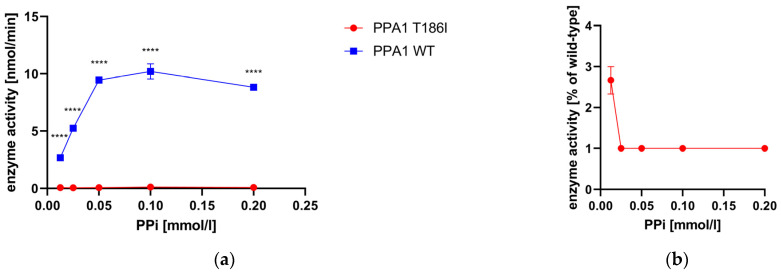
Enzyme activity measurement of recombinant PPA1 variant and wild type. (**a**) Enzyme activity of PPA1 variant and wild type in nmol/min at different PPi concentrations ranging from 0 to 0.2 mM. Two independent measurements were performed (number of measurements: PPA1 variant *n* = 6, wild type: *n* = 6). (**b**) Enzyme activity of the PPA1 variant in % of that of the wild type. (**a**,**b**) Error bars show the standard error of the mean (SEM). Abbreviations: 2-DG, 2-deoxy-D-glucose; WT, wild type; **** *p* < 0.0001.

The recombinant PPA1 variant and wild type were adjusted with Ponceau S staining and western blotting (Figure S3). The enzyme activity of the PPA1 variant and wild type was determined at different PPi concentrations (0–0.2 mM) (Figure 5). The PPA1 variants show reduced enzyme activity at 1–3% that of the wild type (Figure 5).

### 3.7. Extracellular Flux Assay of Primary Human Skin Fibroblasts

**Figure 6:**
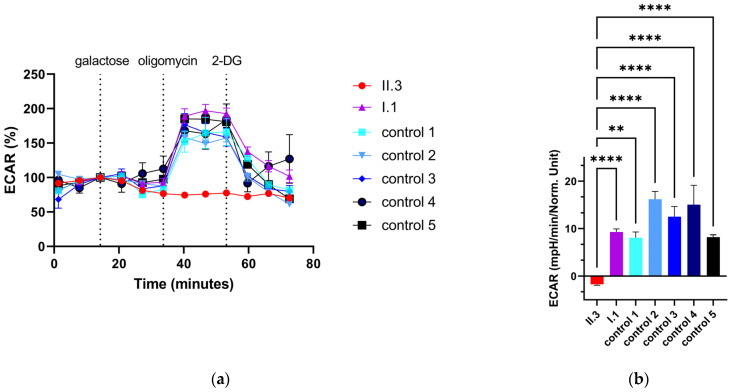
ECAR with galactose in fibroblasts. (**a**) ECAR in % from two independent measurements (number of measurements: affected individual (II.3): n = 13, heterozygous father (I.1): *n* = 15, control 1 *n* = 8, control 2 *n* = 7, control 3 *n* = 13, control 4 *n* = 4, control 5 *n* = 7). (**b**) ECAR in mpH/min/Norm. Unit after the addition of 5 µM oligomycin. Norm. Units illustrate normalized extracellular acidification rate. Bar chart shows galactolytic capacity of the cells. (**a**,**b**) Error bars show the standard error of the mean (SEM). ** *p* < 0.01, **** *p* < 0.0001.

Fibroblasts were grown on galactose for 1 week prior to extracellular flux analysis and seeded on Seahorse plates in starving medium without glucose, galactose or sodium pyruvate. During the assay, a basal value was determined, followed by the addition of galactose, and then oligomycin and finally 2-deoxy-D-glucose (2-DG) (Figure 6). After the addition of oligomycin, a substantial extracellular acidification rate (ECAR) was observed in the fibroblasts of the heterozygous father and controls, indicating elevated galactolytic activity in the cells. In contrast, the fibroblasts of the affected individual (II.3) did not show any galactolytic activity under galactose conditions (Figure 6).

### 3.8. Determination of the Nucleotide Content in Primary Human Skin Fibroblasts

The evaluation of the nucleotide content in the fibroblasts of the affected individual (II.3) compared to that in the fibroblasts of the heterozygous father did not reveal any abnormalities (Figure S4 and Table S4).

## 4. Discussion

The cytosolic pyrophosphatase PPA1 hydrolyses PPi to two molecules of Pi, which are utilized for cellular phosphate homeostasis and drive ATP synthesis. More than 300 metabolic reactions contribute to the overall PPi concentration in the cell, underscoring the importance and ubiquitous role of pyrophosphatases.

We previously reported that impairment of the mitochondrial PPA1 homologue PPA2 underlies an IMD presenting with (infection-triggered) early infantile or adolescent sudden cardiac death (the latter of which is presumably induced by moderate alcohol consumption). Functional characterization of disease-causing *PPA2* missense variants revealed a range of residual activity of recombinant enzymes ranging from 1 to 50%. No patients harboring biallelic PPA2 LoF variants have been reported to date [5].

In Ppa1 knockout mice, no homozygous offspring (Ppa1^−/−^) were born, indicating embryonic lethality. Heterozygous mice (Ppa1^+/−^) showed a considerable reduction in PPA1 in different tissues [7]. These observations suggest that biallelic LoF in PPA1 might be incompatible with life, as a minimal rate of residual enzymatic activity is required to maintain human metabolism [5].

In order to confirm that the reduced pyrophosphatase activity in the 10,000× *g* supernatant of fibroblasts (Figure 4) was due to PPA1 deficiency, we expressed wild-type and mutant PPA1 in *E. coli* and purified the recombinant proteins. Additionally, the pyrophosphatase activity of recombinant proteins showed a clear decrease for the p.Thr186Ile variant. Like in fibroblasts, a higher residual activity was observed at low PPi concentrations (Figure 5), which might indicate that the variant is still sufficiently active under steady-state metabolic conditions. On the other hand, a PPi challenge, e.g., from the diet, might be risky.

The two affected individuals with PPA1 deficiency described in the present study showed fluctuating, but intermittently elevated, metabolites of galactose metabolism. Interestingly, galactose was only initially elevated in the neonatal and early infantile period. Routinely evaluated metabolites of galactose metabolism indicated galactose-1-phosphate as a potential biomarker. Dietary intervention in the form of a galactose-restricted diet (1/3 formula, 2/3 soy milk) in the affected boy at 2 months of age led to a decrease in galactose-1-phosphate levels to normal values. The increase in galactose levels in food (2/3 formula, 1/3 soy milk) resulted again in elevated galactose-1-phosphate. A certain degree of the fluctuation observed in young adolescence might be explained by variable galactose content in the diet; however, this would not lead to an increase above the normal range in healthy individuals and was also not seen in the heterozygous parents or the heterozygous sibling.

Measurements of galactose metabolism were performed in fibroblasts of one of the affected individuals and revealed a clear impairment (Figure 6). The relationship between PPA1 function and galactose metabolism might be explained by the fact that the degradation of galactose produces additional PPi due to the formation of necessary UDP-glucose. UDP-glucose is synthesized from galactose-1-phosphate and UTP-catalyzed by UDP-glucose pyrophosphorylase 2 (UGP2).

The formation of PPi, and hence the canonical breakdown of galactose, results in elevated galactose and galactose-related metabolites [3].

One of the affected individuals (II.3) had neonatal hyperbilirubinemia requiring phototherapy. Although hyperbilirubinemia is a quite common finding in neonates, it is speculated to be related to PPA1 deficiency. Similar to galactose metabolism, the degradation of bilirubin depends on UDP-glucose. The synthesis of UDP-glucose results in PPi, which inhibits bilirubin metabolism and might therefore be related to the neonatal hyperbilirubinemia in the affected girl [24].

Metabolome analysis revealed deranged metabolites, which are members of galactose, amino acids, fatty acids and nucleotide metabolism. Elevated metabolites from amino acid metabolism, such as N-acetyl-L-glutamate 5-semialdehyde, are involved in glutamate and arginine metabolism. Arginine is synthesized in the urea cycle, which is responsible for the catabolism of nitrogen. N-Acetyl-L-glutamate 5-semialdehyde is a metabolite of glutamate degradation which enters the urea cycle for arginine production. Argininosuccinate is synthesized from citrulline, which leads to the formation of PPi [25,26]. In support of these findings, Yin et al. also showed that in *Ppa1*-knockout adipocytes, there was a significant alteration in amino acid and lipid metabolism levels in response to PPA1 deficiency. Effects on beta-oxidation might be reflected by the elevated mid-chain fatty acid 8-[(aminomethyl)sulfanyl]-6-sulfanyloctanoic acid in the blood of the two individuals under investigation [7]. Deranged nucleotide metabolism might also be related to impaired galactose metabolism. UDP-glucose and UPD-galactose are utilized in many biosynthetic pathways, including nucleotide and galactose metabolism. However, despite abnormalities in the nucleotide-activated sugars UDP-glucose and UDP-galactose, there were no indications of misdirected N-glycosylation (Figure S4) [27]. In general, metabolome analysis of dried blood spots revealed that PPA1 deficiency can not only lead to the derangement of the galactose metabolism and PPi formation but also broader abnormalities in amino acid, fatty acid and nucleotide metabolism. The latter is not surprising, since PPi is formed in numerous reactions within different pathways.

Therefore, we can only speculate on the consequences of PPA1 deficiency and the development of clinical symptoms, which presumably will manifest later in life. Our patients may represent the milder end of the clinical spectrum of PPA1 deficiency. We cannot exclude that—in analogy with PPA2 deficiency—rapid life-threatening metabolic derangement could be triggered by secondary stressors that are yet to be determined [5]. In order to avoid any unnecessary PPi sources, we advised the affected individuals to maintain a moderately galactose-restricted diet and abstain from galactose-rich dairy products like fresh milk. Further follow-up of the affected individuals of our family and future identification of similar cases will us help to understand the consequences of the metabolic derangements caused by PPA1 deficiency. Neonatal screening for galactosemia might constitute a future prognostic tool to identify at-risk individuals.

In conclusion, we report a new IMD of pyrophosphate hydrolysis due to biallelic variants in *PPA1* that encode a cytosolic pyrophosphatase. Underlying metabolic impairments can be easily recognized via neonatal galactosemia screening. This is the first ever recognition of cytosolic pyrophosphatase deficiency as an IMD.

## Figures and Tables

**Table 1:** Untargeted metabolomics of DBSs of the affected individuals, II.1 and II.3. and the heterozygous father I.1. Metabolome analysis revealed 19 elevated metabolites in both affected individuals. (**a**) Six metabolites, indicated by “+”, take part in pathways which lead to PPi formation. The remaining seven metabolites were not directly related to PPi-producing pathways and are indicated by “−”. (**b**) Six metabolites were not found in any known metabolic pathway. These metabolites may result from nutrition, muscle degradation, blood regulation or protein degradation.

Elevated Metabolites in DBS of the Two Affected Siblings and the Heterozygous Father							
**(a)**							
**Compound**	**I: 1 (Z-score)**	**II: 1 (Z-score)**	**II: 3 (Z-score)**	**Affected Pathway**	**HMDB ID**	**KEGG-ID**	**PPi formation**
Galactose-1-phosphate	0.569	6.475	5.690	Galactose Metabolism	HMDB0000645	map00052	+
Isobutyrylglycine	1.355	2.823	6.566	Valine, Leucine and Isoleucine Degradation	HMDB0000730	map00280	−
Aminoadipic acid	0.626	2.458	4.823	Lysine Metabolism	HMDB0000510	map00310	−
8-[(Aminomethyl)sulfanyl]-6-sulfanyloctanoic acid	1.561	3.116	3.964	Fatty Acid Synthesis/Beta-Oxidation	HMDB0013639	map00071	−
Imidazole acetol-phosphate	−0.328	2.325	4.607	Histidine Metabolism	HMDB0012236	map00340	−
N-Acetylglutamine | Glycyl-Hydroxyproline	0.700	3.434	2.965	Glutamate Metabolism	HMDB0006029 | HMDB0011173	map00250	+
Galactitol	−0.624	3.156	2.812	Galactose Metabolism	HMDB0000107	map00052	+
N6-Acetyl-L-lysine	0.822	2.990	2.691	Lysine Metabolism	HMDB0000206	map00310	−
Portulacaxanthin II	−0.102	2.811	2.947	Betalain Metabolism	HMDB0012281	map00965	−
2-Hexaprenyl-6-methoxy-1,4-benzoquinol	−0.845	3.064	2.234	Ubiquinone and other Terpenoid-quinone Biosynthesis	HMDB0012148	map00130	+
7-Sulfocholic acid	−1.741	2.289	2.982	Bile Acid Synthesis	HMDB0002421	map00120, map00121	−
Pyrimidine	0.887	2.069	2.489	Pyrimidine Metabolism	HMDB0003361	map00240	+
N-Acetyl-L-glutamate 5-semialdehyde	0.247	2.002	2.114	Arginine and Proline Metabolism	HMDB0006488	map00330	+
**(b)**							
**Compound**	**I: 1 (Z-score)**	**II: 1 (Z-score)**	**II: 3 (Z-score)**	**Affected Pathway**	**HMDB ID**	**KEGG-ID**	**PPi formation**
Vanilpyruvic acid	−2.226	4.723	2.692	Nutrition	HMDB0011714	NA	−
Methionyl-Arginine	−0.911	2.863	3.539	Incomplete Protein Degradation	HMDB0028967	NA	−
Prolyl-Tryptophan	1.498	3.010	2.379	Incomplete Protein Degradation	HMDB0029028	NA	−
Hemorphin-4	−0.352	2.233	2.422	Blood Regulation	HMDB0059788	NA	−
Tyramine-O-sulfate	−0.811	2.285	2.159	Nutrition	HMDB0006409	NA	−
3-Methylhistidine	1.449	2.183	2.221	Muscle Degradation	HMDB0000479	NA	−

## Data Availability

The herein published data will be made available in accordance with the relevant ethical standards and legal guidelines. We will supply our data and material upon request to the corresponding author.

## Supplementary Materials

The following supporting information can be downloaded at: https://www.mdpi.com/article/10.3390/metabo13111141/s1, Table S1: Galactose-1-phosphate (Gal-1-P) levels in blood of the two affected individuals and heterozygous family members. Table S2: targeted metabolic findings in affected individual II.1. Table S3: targeted metabolic findings in affected individual II.3. Table S4: determination of nucleotide content in cell pellets. Figure S1: Sanger sequencing of PPA1 in the affected individuals (II.1, II.3) and heterozygous family members (I.1, I.2, II.2). Figure S2: Western blot of 10,000× *g* supernatant and whole-cell lysates. Figure S3: protein adjustment for enzyme activity measurement of recombinant PPA1 variant and wild type. Figure S4: determination of nucleotide content in cell pellets.
